# Effect of herba centellae on the expression of HGF and MCP-1

**DOI:** 10.3892/etm.2013.1146

**Published:** 2013-06-06

**Authors:** ZHU ZHANG, GUANGCE WANG, JIWEI MA, HAOFEI LIU, XITAO ZHANG, GUANGLING ZHU

**Affiliations:** Department of Nephropathy, The First Affiliated Hospital, Henan University of Traditional Chinese Medicine, Zhengzhou, Henan 450000, P.R. China

**Keywords:** herba centellae, tubulointerstitial fibrosis, hepatocyte growth factor, monocyte chemotactic protein-1

## Abstract

The aim of this study was to explore the effects of herba centellae on protein and mRNA expression of hepatocyte growth factor (HGF), and monocyte chemotactic protein-1 (MCP-1) in renal tubulointerstitial fibrosis (TIF). A unilateral ureteral obstruction (UUO) model was established in 50 male Sprague Dawley (SD) rats. Blood samples were collected and the blood urea nitrogen (BUN) levels, serum creatinine (Scr), alanine aminotransferase (ALT) and aspartate aminotransferase (AST) levels were measured. Immunohistochemistry (IHC) detected the localization and expression levels of HGF and MCP-1. In addition, quantitative polymerase chain reaction (qPCR) detected the mRNA expression of HGF and MCP-1. Thirty rats were used to prepare the rat serum containing drug by cell culture, and qPCR and immunocytochemistry (ICC) were performed to examine the mRNA and protein expression of HGF and MCP-1. MCP-1 and its mRNA expression was significantly higher in rat renal interstitium of the UUO group and cells of the transforming growth factor-β_1_ (TGF-β1) stimulation group compared with that of the control group (P<0.01). MCP-1 and its mRNA expression in the drug intervention group were significantly reduced compared with that of the UUO model group (P<0.01). However, MCP-1 and its mRNA expression in the high-dose herba centellae group was significantly lower compared with that of the low-dose herba centellae group (P<0.01). Furthermore, HGF and its mRNA expression significantly increased in the drug intervention group (P<0.01), and expression in the high-dose group was significantly higher compared with that of the low-dose group (P<0.01), but similar to that of the fosinopril group (P>0.05). The levels of BUN decreased in the drug intervention group; however, no significant differences were determined in Scr, ALT and AST levels. Herba centellae may have inhibited MCP-1 and its mRNA expression through the upregulation of HGF and its mRNA expression, in order to achieve resistance to TIF without showing evident hepatorenal toxicity.

## Introduction

Tubulointerstitial fibrosis (TIF) is a common process, it is the main pathological characteristic of end stage renal disease (ESRD) (1), and is the result of chronic and persistent renal damage. Injured tissues are characterized by excessive proliferation of the extracellular matrix (ECM) due to chronic and persistent damage, subsequently resulting in fibrosis or sclerosis (2,3). Iwano *et al* determined that ∼42% of renal TIF derived from renal tubular epithelial cells (4). The renal tubule connects the glomerulus and renal interstitium. Renal tubular epithelial cells are directly involved in renal tubular atrophy, expansion of the renal tubule cavity and are related to ECM proliferation. Therefore, renal tubular epithelial cells have a significant impact on the initiation of TIF, and the development and inhibition of TIF is important to impede the processes of renal disease. The mechanisms of TIF involve various renal inherent cells, cytokines and bioactive substances, and an imbalance in the cytokines that inhibit or facilitate fibrosis is important.

Hepatocyte growth factor (HGF) is a cytokine indicated to be anti-fibrotic and may inhibit TIF; however, the mechanism of anti-TIF has not been fully understood. Monocyte chemoattractant protein-l (MCP-1) is an important medium for monocyte/macrophage infiltration and a principle cytokine that may induce TIF (5,6). The prevention and delayed occurence of TIF is a worldwide difficulty. Therefore, studies aim to determine novel therapeutic targets and develop drugs with strong pertinence, efficacy and fewer side effects so as to prevent and treat patients with TIF.

Modern clinical and pharmacological studies have demonstrated that herba centellae contains triterpenoids, including asiaticoside, madecassoside, madecassic acid and asiatic acid (7–10). Herba centellae may inhibit proliferation of the ECM and maintain the fibre composition by decreasing aminotransferase activity, reducing acid mucopolysaccharide and collagen, and inhibiting the expression of transforming growth factor-β_1_ (TGF-β1). Expression of TGF-β1 may be inhibited by enhancing Smad expression, which blocks proliferation of the fibroblasts and prevents inflammation and proliferation. Furthermore, herba centellae may promote normal granulated tissue formation, activate epithelial cells, accelerate wound healing and inhibit fibroblast proliferation and collagen formation, thus inhibiting excessive proliferation of the connective tissue matrix and maintain fibre composition. Herba centellae is not expensive and has significant efficacy for fibrotic diseases including the treatment of scarring from burns and scleroderma, with no toxic effects. Therefore, herba centellae is advantageous for the treatment of fibrotic diseases. Previous studies have demonstrated that herba centellae may reduce the expression of renal tissue connective tissue growth factor (CTGF) and α-smooth muscle actin (α-SMA) in unilateral ureteral obstruction (UUO) rats, inhibit the expression of TGF-β1 in tubular epithelial cells *in vitro*, and maintain the expression of bone morphogenetic protein-7 (BMP-7). Thus, herba centellae may reduce tubulointerstitial damage and exhibit anti-TIF effects. However, whether herba centellae may promote ECM degradation requires further study. In addition, herba centellae may regulate the expression of HGF and MCP-1 in ECM degradation and prevent and delay TIF; however, the potential mechanisms have not been studied.

In the present study, based on previous work model animals with tubulointerstitial damage were established *in vivo* and *in vitro*. The protein and mRNA expression of HGF and MCP-1 in the renal tissue of these models was determined using qPCR, immunohistochemistry (IHC) and immunocytochemistry (ICC). This study aimed to identify significant mechanisms for the prevention and delay of TIF.

## Materials and methods

### General data

Eighty male Sprague-Dawley rats at 6–8 weeks old (weight, 200±20 g) were supplied by the Henan animal experiment center (Henan, China). This study was carried out in strict accordance with the recommendations in the Guide for the Care and Use of Laboratory Animals of the National Institutes of Health. The animal use protocol was reviewed and approved by the Institutional Animal Care and Use Committee (IACUC) of The First Affiliated Hospital, Henan University of Traditional Chinese Medicine (Zhengzhou, Henan, China). The animal model was established in 50 rats among which, 10 rats were randomly selected as the control group and the remaining 40 rats were evenly and randomly divided into the model group, high-dose herba centellae, low-dose herba centellae and fosinopril groups. The rats were sacrificed 21 days following drug application and the kidneys were dissected. Blood samples were collected from the abdominal aorta for biochemical testing. An additional 30 rats were used to prepare the rat serum containing drug by cell culture. The epithelial cell strain-NRK52E of proximal convoluted tubule from normal rats was a gift from Professor Xueqing of Zhongshan University (Guangzhou, Guangdong, China).

### Animal model establishment

Rats from the surgery group were anesthetized by intraperitoneal injection using 10% chloral hydrate (0.3 ml/100 g). An incision was made on the left abdomen to expose the left kidney and ureter, which was subsequently ligated and cut from the proximal inferior pole of the left kidney. The control group did not undergo UUO surgery but followed a similar method to the surgery group.

### Tissue sample preparation

Rats were anesthetized as previously mentioned and the abdominal cavity was exposed in the middle. Blood samples were collected from the abdominal aorta and the left kidney was dissected. A quarter of the renal tissue was fixed in 4% paraformaldehyde, dehydrated by gradient alcohol, transparentized using xylol and immersed in paraffin for 3 h at 62°C prior to embeddeding. The expression of HGF and MCP-1 was examined in the tissue sections using hematoxylin and eosin (HE) staining and immunohistochemistry (IHC). The remaining renal tissue was frozen in liquid nitrogen and stored in a freezer (−70°C) for subsequent qPCR analysis.

### IHC-streptavidin-peroxidase (SP)

The paraffin-embedded renal tissue section was cut into 4 *μ*m slides, deparaffinized and rehydrated. The slides were incubated with primary antibodies of rabbit-anti-rat HGF and rabbit-anti-rat MCP-1 (1:200, Wuhan Boster Biological Engineering Co., Ltd., Hubei, China) at 40°C overnight. Then, biotinylated sheep-anti-rabbit IgG (Wuhan Boster Bioengineering Co., Ltd.) was added to the tissue sections for 20 min at 37°C. Diaminobenzidine (DAB)-H_2_O_2_ (Wuhan Boster Biological engineering Co., Ltd.) was used to develop the color and hematoxylin was applied for counterstaining, and the slides were covered for examination. Ten fields of renal tubulointerstitial on each slide were recorded randomly under high magnification (×400) and Taimeng multimedia pathology analysis software (Taimeng Technology Ltd., Chengdu, Sichuan China) was used for image analysis. Yellow or brown granules in the cytoplasm were recorded as a positive signal, and the average optical density (OD) of positive and background staining was measured following chromaticity transformation.

### qPCR

The total RNA of rat renal tissue was extracted by TRIzol (Shanghai Sangon Biological Engineering Technology and Services Co., Ltd., Shanghai, China) and the purity and content was measured by ultraviolet-visible (UV-VIS) spectrophotometer. qPCR was performed according to the method from the real-time kit (Shanghai Sangon Biological Engineering Technology and Services Co., Ltd., Shanghai, China). Total RNA from each group (2 *μ*l) was obtained and reverse transcribed to cDNA. The total volume was 21 *μ*l under the conditions of 70°C for 5 min, 37°C for 5 min, 37°C for 60 min and 70°C for 10 min. The cDNA was used as a template for the PCR reaction with a total volume of 25 *μ*l. The primer of rat HGF was designed as follows: forward: 5′-TACACTCTTGACCCT GACACCC-3′, and reverse 5′-TTTCCCATTGCCACGATA ACA-3′; length of PCR amplified fragment, 378 bp. The primer of rat MCP-1 was designed as follows: forward: 5′-TCTCTGTCATACTGGTCACTTC-3′, and reverse 5′-GGT GTCCCAAAGAAGCTGTAG-3′; length of PCR amplified fragment, 270 bp. The primer of rat glyceraldehyde 3-phosphate dehydrogenase (GAPDH) was designed as follows: forward: 5′-TGACATCAAGAAGGTGGTGA-3′, and reverse 5′-TCATACCAGGAAATGAGCT-3′; length of the PCR amplified fragment, 177 bp. The reaction was carried out over 34 cycles for HGF in the following conditions: 3 min at 95°C, 30 sec at 94°C, 60 sec at 55°C and 60 sec at 72°C. The reaction for MCP-1 was carried out over 33 cycles for 3 min at 95°C, 30 sec at 94°C, 60 sec at 55°C and 60 sec at 72°C. The reaction for GAPDH was carried out over 30 cycles for 2 min at 94°C, 45 sec at 94°C, 30 sec at 60°C and 1 min at 72°C. The product of the PCR was added to 2% agarose and run in gel electrophoresis. The DNA fragments were then set in the gel image analysis system (UVP Inc., PA, USA) to scan the absorbance. GAPDH was considered as internal reference, and the ratio of the target gene absorbance and GAPDH absorbance was recorded as the relative target gene expression.

### Preparation of rat serum containing drug

Thirty healthy SD rats were randomly divided into the herba centellae, fosinopril and control groups. According to the Traditional Chinese medicine pharmacological research methodology, 10 times herba centellae granules of adult dose/kg were dissolved in triple distilled water and gavaged with a dose of 2 ml/(100 g/d) (0.25 g raw herba centellae/m; Jiangyin Tianjiang Pharmaceutical Co., Ltd., Jiangsu, China) twice per day for three days. The interval between administration on the last day was 2 h and the rats were sacrificed 1–2 h following the final dose. Blood samples were collected from the abdominal aorta and the serum was isolated. The complement of serum with drug was inactivated in a water bath for 30 min at 56°C, followed by 0.22 *μ*m filtration and sterilization and then stored in the freezer (−70°C) for further use. In the fosinopril group (Sino-US Shanghai Bristol-Myers Squibb Pharmaceutical Co., Ltd., Shanghai China) samples were prepared as previously mentioned and in the control group physiological saline was used to gavage the rats.

### Cell culture

NRK52E cells were cultured in Dulbecco’s modified Eagle’s medium (DMEM)/nutrient mixture F-12 media (contained 100 U/ml penicillin, 100 ttg/ml streptomycin) with 10% fetal bovine serum (FBS) at 37°C and 5% CO_2_. Following passage, cells were inoculated into six 25 cm^2^ culture flasks. Moreover, cells were synchronized in the non-serum media for 12 h and divided into five groups stochastically when they reached 80% confluence. In the control group (DZ) cell culture was determined as DMEM/nutrient mixture F-12 + 10% blank serum; in the TGF-β_1_ (PeproTech Inc., NJ USA) stimulation group (T) as DMEM/F-12 (with 10 ng/ml TGF-β_1_) + 10% blank serum; in the low-dose group as DMEM/F-12 (with 10 ng/ml TGF-β_1_) + serum with 2.5% herba centellae; in the high-dose group as DMEM/F-12 (with 10 ng/ml TGF-β_1_) + serum with 10% herba centellae; and in the fosinopril group (M) as DMEM/F-12 (with 10 ng/ml TGF-β_1_) + serum with 10% fosinopril. In addition, blank serum was replenished to 10% serum concentration in each group. The cells were cultured for 48 h in 5% CO_2_ at 37°C. Following cultivation, the HGF and MCP-1 protein and mRNA expression levels were assayed by qPCR and ICC methods. The experiments were repeated 10 times independently.

### ICC

Following 48 h of cell stimulation with drugs, the sterile cover glass for cell culture was placed over a six-well plate. The culture media were drained away and the six-well plate was washed twice with phosphate-buffered saline (PBS), and cells were fixed with 4% paraformaldehyde for 15 min at 4°C. The cover glass was removed and washed with 0.01 M PBS (pH=7.4) three times (3–5 min each time). Following inactivation of endogenous peroxidase, the slides were incubated with primary antibodies (rabbit IgG, 1:100) at 4°C overnight. In addition, the horseradish peroxidase (HRP)-labeled anti-rabbit IgG was added to the cover glass for 30 min at 37°C. DAB-H_2_O_2_ was utilized to develop the color and hematoxylin was applied for counterstaining. The slides were covered for examination. Ten fields of renal tubulointerstitial on each slide were recorded randomly by Olympus microscope (Japan) and Taimeng multimedia pathology analysis software (Taimeng Technology Ltd, Chengdu, Sichuan, China) was used for image analysis. Yellow or brown granules in the cytoplasm were recorded as a positive signal, and the average optical density (OD) of positive and background staining were measured following chromaticity transformation. Darker staining correlated with a higher OD level.

### Fluorescent quantification (FQ) qPCR

Following collection, the cells were washed with PBS and the total RNA was extracted following the RNAiso reagent (Takara Bio, Inc., Japan) protocol. RNA concentration was calculated based on the A260/A280 value measured by an ultraviolet spectrophotometer. HiFi-MMLV-cDNA First-Strand cDNA Synthesis Kit (Shanghai Sangon Biological Engineering Technology & Services Co., Ltd.) was utilized to synthesize the first-strand cDNA, and the PCR kit was used to perform the PCP reaction. The primers used were: MCP-1: forward: 5′-TACAAGAGAATCACCAGCAGCA-3′, and reverse: 5′-TACAAGAGAATCACCAGCAGCA-3′; product length, 90 bp; HGF: forward: 5′-TTCCCGTTGTGAAGGAGAT ACT-3′, and reverse: 5′-ACCATCCACCCTACTGTTGTTT-3′; product length, 129 bp; GAPDH: forward: 5′-TGACATCAA GAAGGTGGTGA-3′; and reverse: 5′-TCATACCAGGAAA TGAGCT-3′; product length, 177 bp. The reaction occurred for 2 min at 50°C, followed by 40 cycles of 10 min at 95°C, 15 sec at 95°C and 1 min at 60°C. The end of the reaction included 15 sec at 95°C and 15 sec at 60°C. The fluorescent signal was obtained during the annealing process. The results from FQ qPCR were represented by the Ct value, which was the number of cycles in each reaction when the fluorescent signal reached the set threshold. The Ct value was calculated as: average Ct value of a gene-Ct value of GAPDH.

### Statistic analysis

Data were presented as means ± SD. Statistical significance was performed using SPSS software version 18.0 (SPSS, Inc., Chicago, Il USA). Comparison between groups was examined using the one-way analysis of variance (ANOVA). P<0.05 indicates a statistically significant difference.

## Results

### Protein expression of HGF and MCP-1 in rat renal tissue

**Figure 1.**
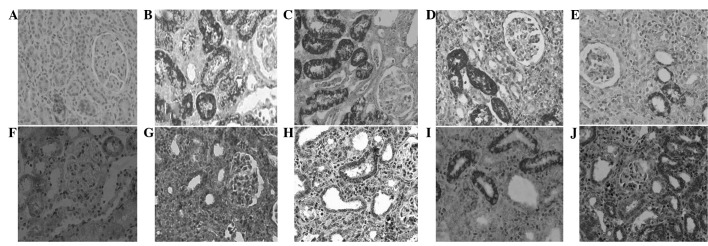
Immunohistochemistry-streptavidin-peroxidase (IHC-SP) of the kidneys in each group (×400). The expression of monocyte chemotactic protein-1 (MCP-1) in (A) the control; (B) model; (C) low-dose; (D) high-dose; and (E) fosinopril groups. The expression of hepatocyte growth factor (HGF) in the (F) control; (G) model; (H) low-dose; (I) high-dose; and (J) fosinopril groups.

The IHC demonstrated a low expression of HGF in renal tubulointerstitial cells of the control group; however, following UUO surgery HGF was mainly expressed in the cytoplasm of the renal tubular epithelial cells. In addition, there was a low expression of HGF in renal tubulointerstitial cells in the model group. However, the expression of HGF had significantly increased in the drug intervention group (P<0.01) compared with that of the control group. Expression in the high-dose group was significantly higher compared with that of the low-dose group (P<0.01), but similar to that of the fosinopril group (P>0.05). MCP-1 was predominantly expressed in the cytoplasm of renal tubular epithelial cells; however, a low expression was indicated in the renal tissue, and no expression was observed in the glomerulus and renal interstitial cells in the control group. MCP-1 expression in renal tissue significantly increased 21 days following surgery in the model group compared with that of the control group, and a particularly significant increase was demonstrated in the medulla tubule epithelial cells (P<0.01). Statistical analysis determined that MCP-1 expression in the drug intervention group was significantly reduced compared with that of the UUO model group (P<0.01). However, expression of MCP-1 in the high-dose group was significantly lower compared with that of the low-dose group (P<0.01), and that of the fosinopril group, (P>0.05; Fig. 1, Table I).

### mRNA expression of HGF and MCP-1 in rat renal tissue

**Figure 2.**
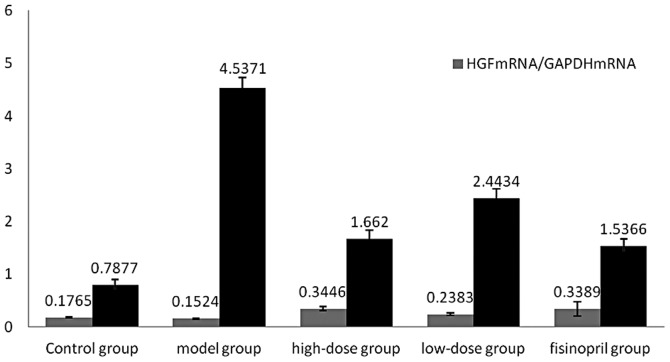
Levels of mRNA expression of hepatocyte growth factor (HGF) and monocyte chemotactic protein-1 (MCP-1) in unilateral ureteral obstruction (UUO) renal tissue of each group.

**Figure 3.**
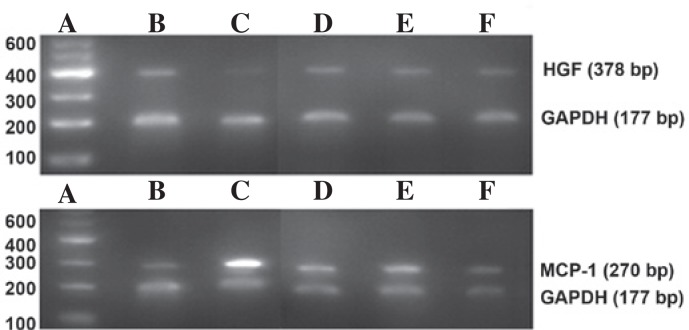
Detection of mRNA expression of hepatocyte growth factor (HGF) and monocyte chemotactic protein-1 (MCP-1) in unilateral ureteral obstruction (UUO)-renal tissue by reverse transcription-polymerase chain reaction (RT-PCR). (A) Marker; and (B) control; (C) model; (D) high-dose; (E) low-dose; and (F) fosinopril groups.

The results of the qPCR demonstrated that mRNA expression of HGF had significantly increased in the drug intervention group (P<0.01) compared with that of the control group. In addition, a low mRNA expression of HGF was indicated in the model group, whereas mRNA expression was significantly higher in the high-dose group compared with that of the low-dose group (P<0.01), and similar to that of the fosinopril group (P>0.05). The mRNA expression of MCP-1 in renal tissue significantly increased in the model group compared with that of the control group which indicated marginal mRNA expression of MCP-1. Statistical analysis determined that mRNA expression of MCP-1 in renal tissue on the obstruction side in the drug intervention group was significantly reduced compared with that of the UUO model group (P<0.01). Moreover, MCP-1 mRNA expression in the high-dose group was significantly lower than that of the low-dose group (P<0.01), but marginally higher than that of the fosinopril group (P>0.05; Table II, Figs. 2 and 3).

### Biochemical index changes

The blood urea nitrogen (BUN) levels significantly increased in the model group and a change in the level of BUN was evident in the drug intervention group. The BUN level was significantly lower in the low-dose group (P<0.05) as well as the high-dose and fosinopril groups (P<0.01), compared with that of the control group. An increase in BUN levels in the control group was indicated; however, it was the smallest increase among all the groups. The level of serum creatinine (Scr) was in a normal range for all the rats, and the changes of aspartate aminotransferase (AST) and alanine aminotranferase (ALT) levels were not significant among the groups (P>0.05; Table III).

### HGF and MCP-1 expression induced by TGF-β1 in NRK52E cells by ICC

**Figure 4.**
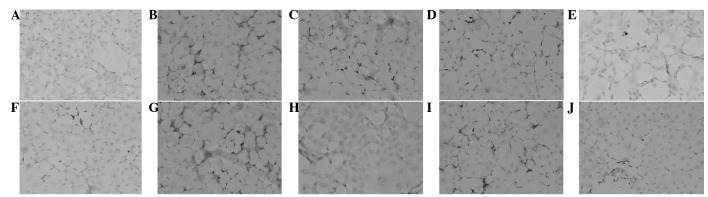
Immunocytochemistry (ICC) in each group (SV staining, ×400). Hepatocyte growth factor (HGF) expression in the (A) transforming growth factor-β_1_ (TGF-β_1_) ; (B) fosinopril; (C) high-dose; (D) low-dose; and (E) in control groups. Monocyte chemotactic protein-1 (MCP-1) expression in the (F) control; (G) TGF-β1; (H) high-dose; (I) low-dose; and (J) fosinopril groups.

ICC demonstrated a marginal expression of HGF in the control group. In the TGF-β1 group the expression of HGF was mainly located in the cytoplasm of NRK52E cells. In the drug intervention group, the expression of HGF had significantly increased compared with that of the control group (P<0.01). Additionally, the expression of HGF in the high-dose group was significantly higher than that of the low-dose group (P<0.01); but similar to that of the fosinopril group (P>0.05). ICC determined the expression of MCP-1 which was indicated in cells with brown cytoplasm. Marginal expression of MCP-1 was identified in the control group. However, a higher expression was indicated in the TGF-β1 stimulation group as the cells characterized by brown cytoplasm (shown as darker staining) transformed into fibroblasts. The expression of MCP-1 in the high-dose group was significantly lower than that of the low-dose group (P<0.01), and marginally lower than that of the fosinopril group (P>0.05; Table IV, Fig. 4).

### HGF and MCP-1 mRNA expression by FQ qPCR

**Figure 5.**
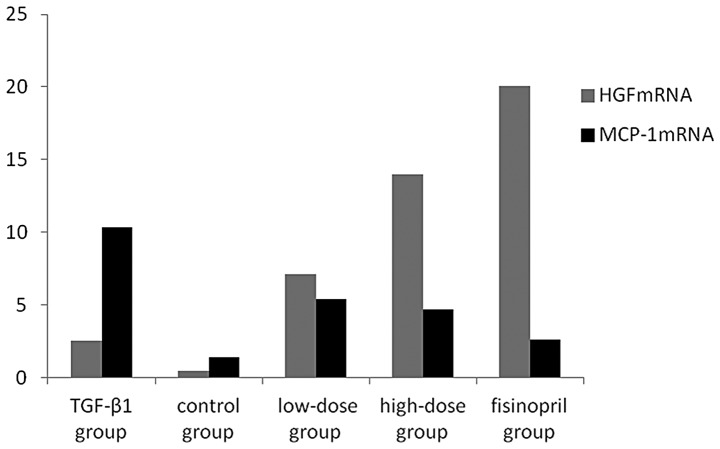
Levels of mRNA expression of hepatocyte growth factor (HGF) and monocyte chemotactic protein-1 (MCP-1) in the NRK52E cells of each group.

**Figure 6.**
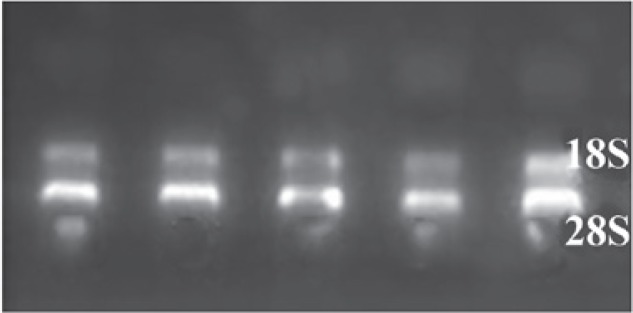
Gel electrophoresis of fluorescent quantification, quantitative polymerase chain reaction (FQ qPCR) products in NRK52E cells of the (A) transforming growth factor-β_1_ (TGF-β_1_); (B) control; (C) high-dose; (D) low-dose; and (E) fosinopril groups.

FQ qPCR indicated marginal mRNA expression of HGF in the control group. Administration of high- and low-doses of herba centellae may have promoted NRK52E cells to stimulate TGF-β1, which subsequently induced mRNA expression of HGF. The mRNA expression of HGF in the high-dose group was significantly higher than that of the low-dose group (P<0.05), and similar to that of the fosinopril group (P>0.05). The mRNA expression of MCP-1 was significantly higher in the TGF-β1 stimulation group compared with that of the drug intervention groups (P<0.01), in a dose-dependent manner. FQ qPCR determined no significant differences between the high-dose group and the fosinopril group (P>0.05, Figs. 5 and 6, Table V).

## Discussion

TIF is a tubular injury resulting from inflammatory cell infiltration, fibroblast proliferation, and conversion to fibrocytes which are caused by a variety of kidney damage factors (11). Progressive and irreversible renal damage is the result of TIF. Bohle *et al* (12) demonstrated that renal failure was closely correlated with TIF, which was an etiological factor-independent process. Studies worldwide have achieved great progress in investigating TIF. TIF is characterized by fibroblast proliferation and excess of the ECM including collagen I, II, III, IV, fibronectin and laminin, which accumulates in the renal interstitium. A previous theory was that fibrosis could not be reversed. However, a large number of experiments have indicated that fibrosis may be reversed at a certain stage. Therefore, the prevention and delay of TIF initiation and development, is one of the predominant measures for retarding the deterioration of renal function. However, the mechanism of TIF has yet to be fully understood. A previous study (13) demonstrated that the mechanism of TIF may include transdifferentiation of renal tubular epithelial cells (TEC). It demonstrated that TEC may transdifferentiate into myofibroblasts which, subsequently promoted fibrosis formation by EMC synthesis. In addition, the study showed that cytokines and growth factors are important for TIF initiation and development. The growth factors [TGF-β, connective tissue growth factor (CTGF), platelet-derived growth factor (PDGF)], MCP-1, Angiotensin II (Ang II) and endothelin-1 (ET-1) promote TIF initiation, and TGF-β was identified as the most important promoter. Furthermore, interferon-γ (IFN-γ), HGF, bone morphogenetic protein-7 (BMP-7) and decorin demonstrated the ability to delay TIF initiation. Moreover, the study showed there is monocyte/macrophage infiltration in the renal interstitium at the early stages of TIF in animal models and in a clinical setting. Active monocyte/macrophage infiltration is an important TIF-promoting factor. Zeisberg *et al* determined that cell proliferation and apoptosis included fibroblast proliferation and TEC apoptosis. Additionally, TIF mechanisms may include dysfunction of the EMC hydrolase system (13).

Renal damages induced by UUO include atrophy and apoptosis of renal tubule cells, and TIF and mild pathologic changes of the glomerulus, which is considered an ideal TIF model. TIF is a common result of chronic and persistent renal diseases and is the main pathological feature of end stage renal disease (ESRD). ESRD is characterized by the degeneration, atrophy and disappearance of TEC, the infiltration of renal interstitial lymphatic mononuclear cells and excessive accumulation of the ECM (14). The pathogenesis of TIF has not been completely understood, but studies have shown that the degree of renal tubulointerstitial damage was positively correlated with renal function, and a prognosis may be determined when compared with the glomerulus. Therefore, pathological changes of TIF are a significant index to determine the degree of reduced renal function and a prognosis of the disease.

HGF, a TIF-inhibiting pleiotropic and polypeptide cytokine, may block the TGF-β1/Smad signaling pathway by suppressing the expression of TGF-β1, and in turn reduce the levels of CTGF (15). HGF may prevent the epithelial mesenchymal transition (EMT) of kidney tubules induced by TGF-β1, reverse the phenotypic transformation caused by TGF-β1, restart the expression of E-cadherin, inhibit the expression of the marker of interstitial fibrosis α-SMA, and restore the expression of vimentin and fibronectin (16). HGF has a potent antifibrotic effect. In many animal models with TIF, injection of exogenous recombinant HGF may block the occurrence of fibrosis, inhibit scar formation, and improve renal function (17).

Studies have demonstrated that MCP-1 mediate lysosomes to release and produce oxygen-free radicals, thus promoting mononuclear macrophages to express TGF-β1. Therefore, thickening of the glomerular basement membrane and accumulation of the ECM was aggravated, resulting in glomerulosclerosis and interstitial fibrosis (18). In the anti-glomerular basement membrane (GMB) nephritis model, macrophage infiltration increased in the glomerulus along with elevated MCP-1 expression, while the macrophages decreased following anti-MCP-1 antibody treatment (19). An enhanced number of macrophages and expression of MCP-1 has been determined in the glomerulus and renal tubulointerstitial of the UUO model (20). In addition to the recruiting macrophage, MCP-1 may also be important in fibrosis. The present study demonstrated that MCP-1 may reduce the accumulation of macrophages in renal tissue and limit renal damage by blocking MCP-1 expression (9). Ranieri *et al* (10) indicated that MCP-1 may stimulate TCE culture *in vitro* to express interleukin-6 (IL-6) and intercellular adhesion molecule-1 (ICAM-1) in a time- and dose-dependent manner. Furthermore, IL-6 is a cytokine causing mesangial cell proliferation and TEC atrophy; therefore, MCP-1 is important for the formation of TIF.

The present study applied herba centellae granules in a clinical setting for the treatment of chronic kidney disease (CKD). Herba centellae may reduce proteinuria to some extent, and improve renal function; however, the mechanism is not clear. The UUO model and TGF-β1 stimulation experiments *in vitro,* indicated that herba centellae may upregulate HGF and its mRNA expression in rat renal interstitium, and downregulate MCP-1 and its mRNA expression. Therefore, this delayed the progress of TIF in a dose-dependent manner, determining that a high drug dose had a stronger effect on the promotion of HGF and inhibition of MCP-1, compared with a low drug dose and is similar to that of the fosinopril group (P>0.05). Both low- or high-doses of herba centellae may have promoted NRK52E cells to express HGF and its RNA. The expression in the high-dose group was significantly higher compared with that of the low-dose group (P<0.05). In addition, MCP-1 and its mRNA expression in the drug intervention group was significantly reduced compared with that of the TGF-β1 stimulation group (P<0.01) in a dose-dependent manner. Moreover, MCP-1 and its mRNA expression in the high-dose group was similar to that of the fosinopril group (P>0.05). The *in vivo* and *in vitro* experiment results are consistent with previous hypotheses, and are in accordance with other relevant studies (21–23). This study demonstrated that herba centellae reduced the levels of BUN, to some extent, in UUO rats without causing a significant change in Scr, ALT and AST expression. Therefore, this study determined that herba centellae may inhibit MCP-1 and its mRNA expression through the upregulation of HGF and its mRNA expression, in order to achieve resistance to TIF without showing evident hepatorenal toxicity.

## Figures and Tables

**Table I.** Comparison of HGF and MCP-1 expression in renal tissue of each group (mean ± SD).

Groups	n	HGF	MCP-1
Control	10	0.1174±0.0296	0.0778±0.0432
Model	10	0.0703±0.0274	0.6545±0.0768^a^
High-dose	10	0.1255±0.0283^a–d^	0.3473±0.0424^a–d^
Low-dose	10	0.1709±0.0521^a,b^	0.4632±0.0156^a,b^
Fosinopril	10	0.2512±0.0659^a,b^	0.2797±0.0348^a,b^

a P<0.01, compared with the control group;

b P<0.01, compared with the model group;

c P<0.01, high-dose group compared with the low-dose group;

d P>0.05, high-dose group compared with the fosinopril group. HGF, hepatocyte growth factor; MCP-1, monocyte chemotactic protein-1.

**Table II.** Comparison of HGF and MCP-1 mRNA expression in renal tissue of each group (mean ± SD).

Groups	n	HGF mRNA/GAPDH mRNA	MCP-1 mRNA/GAPDH mRNA
Control	10	0.1765±0.0121	0.7877±0.1059
Model	9	0.1524±0.0088	4.5371±0.3158^a^
High-dose	9	0.2292±0.0215^a,b^	2.3954±0.1792^a–d^
Low-dose	10	0.1986±0.0149^a,b^	2.9647±0.1476^a,b^
Fosinopril	10	0.3389±0.1340^a,b^	1.5336±0.1340^a,b^

a P<0.01, compared with the control group;

b P<0.01, compared with the model group;

c P<0.01, high-dose group compared with the low-dose group;

d P>0.05, high-dose group compared with the fosinopril group. GAPDH, glyceraldehyde 3-phosphate dehydrogenase; HGF, hepatocyte growth factor, MCP-1, monocyte chemotactic protein-1.

**Table III.** Comparison of biochemical indexes of rats in each group (mean ± SD).

Groups	n	BUN	Scr	AST	ALT
Control	10	6.48±0.53^a^	51.48±2.51	124.14±19.10	29.23±3.02
Model	10	12.51±1.34	61.75±2.85	125.61±13.98	29.42±5.29
High-dose	10	9.27±0.64^a^	58.16±1.37	130.83±10.79	30.55±4.73
Low-dose	10	9.89±0.99^b^	59.91±1.14	126.28±15.00	29.59±5.65
Fosinopril	10	7.89±0.75^a^	51.00±1.96	130.44±16.04	30.27±4.64

a P<0.01, compared with the model group;

b P<0.05, low-dose group compared with the model group. BUN, blood urea nitrogen; Scr, serum creatinine; AST, aspartate aminotransferase; ALT, alanine aminotransferase.

**Table IV.** Comparison of HGF and MCP-1 expression in the cells of each group (mean ± SD).

Group	n	HGF	MCP-1
Control	10	63.903±0.0296	48.026±1.524^a^
TGF-β1	10	35.605±1.0910	146.177±3.694
High-dose	10	117.575±4.1940^a–d^	77.817±5.292^a–d^
Low-dose	10	141.140±3.6910^a,b^	65.115±3.179^a,b^
Fosinopril	10	184.467±3.2900^a,b^	58.221±3.726^a,b^

a P<0.01, compared with the control group;

b P<0.01, compared with the model group;

c P<0.01, high-dose group compared with the low-dose group;

d P>0.05, high-dose group compared with the fosinopril group. TGF-β_1_, transforming growth factor-β_1_; HGF, hepatocyte growth factor, MCP-1, monocyte chemotactic protein-1.

**Table V.** Comparison of HGF and MCP-1 mRNA expression in the cells of each group (mean ± SD).

Groups	n	HGF mRNA/GAPDH mRNA	MCP-1 mRNA/GAPDH mRNA
Control	10	0.4760±0.135^b^	1.357±0.594
TGF-β_1_	10	2.4975±0.826^a^	10.314±0.931^a^
High-dose	10	13.9810±1.584^a–c^	4.712±0.821^a–d^
Low-dose	10	7.1250±1.038^a,b^	5.367±1.187^a,b^
Fosinopril	10	20.0910±2.0138^a,b^	2.631±0.835^a,b^

a P<0.01, compared with the control group;

b P<0.01, compared with the model group;

c P<0.01, high-dose group compared with the low-dose group;

d P>0.05, high-dose group compared with the fosinopril group. GAPDH, glyceraldehyde 3-phosphate dehydrogenase; HGF, hepatocyte growth factor; MCP-1, monocyte chemotactic protein-1.
